# Utilizing minimally purified secreted rAAV for rapid and cost-effective manipulation of gene expression in the CNS

**DOI:** 10.1186/s13024-020-00361-z

**Published:** 2020-03-02

**Authors:** Marshall S. Goodwin, Cara L. Croft, Hunter S. Futch, Daniel Ryu, Carolina Ceballos-Diaz, Xuefei Liu, Giavanna Paterno, Catalina Mejia, Doris Deng, Kimberly Menezes, Laura Londono, Kefren Arjona, Mary Parianos, Van Truong, Eva Rostonics, Amanda Hernandez, Sanford L. Boye, Shannon E. Boye, Yona Levites, Pedro E. Cruz, Todd E. Golde

**Affiliations:** 1 Department of Neuroscience, College of Medicine, University of Florida, Gainesville, Florida USA; 2 Center for Translational Research in Neurodegenerative Disease, College of Medicine, University of Florida, Gainesville, Florida USA; 3 Department of Pediatrics and the Powell Gene Therapy Center, University of Florida, Gainesville, Florida USA; 4 Department of Ophthalmology, University of Florida, Gainesville, Florida USA; 5 McKnight Brain Institute, College of Medicine, University of Florida, Gainesville, Florida USA

**Keywords:** Adeno-associated virus, Capsid pseudo-type, AAV production, Gene delivery, Central nervous system

## Abstract

**Background:**

Recombinant adeno-associated virus (rAAV) is widely used in the neuroscience field to manipulate gene expression in the nervous system. However, a limitation to the use of rAAV vectors is the time and expense needed to produce them. To overcome this limitation, we evaluated whether unpurified rAAV vectors secreted into the media following scalable PEI transfection of HEK293T cells can be used in lieu of purified rAAV.

**Methods:**

We packaged rAAV2-EGFP vectors in 30 different wild-type and mutant capsids and subsequently collected the media containing secreted rAAV. Genomic titers of each rAAV vector were assessed and the ability of each unpurified virus to transduce primary mixed neuroglial cultures (PNGCs), organotypic brain slice cultures (BSCs) and the mouse brain was evaluated.

**Results:**

There was ~ 40-fold wide variance in the average genomic titers of the rAAV2-EGFP vector packaged in the 30 different capsids, ranging from a low ~ 4.7 × 10^10^ vector genomes (vg)/mL for rAAV2/5-EGFP to a high of ~ 2.0 × 10^12^ vg/mL for a capsid mutant of rAAV2/8-EGFP. In PNGC studies, we observed a wide range of transduction efficiency among the 30 capsids evaluated, with the rAAV2/6-EGFP vector demonstrating the highest overall transduction efficiency. In BSC studies, we observed robust transduction by wild-type capsid vectors rAAV2/6, 2/8 and 2/9, and by capsid mutants of rAAV2/1, 2/6, and 2/8. In the in vivo somatic brain transgenesis (SBT) studies, we found that intra-cerebroventricular injection of media containing unpurified rAAV2-EGFP vectors packaged with select mutant capsids resulted in abundant EGFP positive neurons and astrocytes in the hippocampus and forebrain of non-transgenic mice. We demonstrate that unpurified rAAV can express transgenes at equivalent levels to lysate-purified rAAV both in vitro and in vivo. We also show that unpurified rAAV is sufficient to drive tau pathology in BSC and neuroinflammation in vivo*,* recapitulating previous studies using purified rAAV.

**Conclusions:**

Unpurified rAAV vectors secreted into the media can efficiently transduce brain cells in vitro and in vivo, providing a cost-effective way to manipulate gene expression. The use of unpurified virus will greatly reduce costs of exploratory studies and further increase the utility of rAAV vectors for standard laboratory use.

## Background

Adeno-associated virus (AAV) was first discovered in 1965 as a contaminant in adenovirus preparations [1–3]. Further investigation revealed that AAV consists of a single stranded DNA genome within a capsid protein shell and that multiple unique AAV serotypes infect vertebrate species including humans and non-human primates [4–7]. Unlike other viruses such as adenovirus, AAV infection does not cause any known human diseases. Studies of AAV biology led to the cloning of the AAV serotype 2 (AAV2) genome into expression plasmids which revealed that infectious viral particles lacking AAV protein-coding genes could be produced in vitro by supplying these genes in *trans* [8–10]. Together, these findings suggested use of AAV as a promising candidate for gene delivery. Over the next several decades, AAV was extensively engineered by replacing all viral protein-coding sequences with non-viral transgene cassettes. The resulting rAAV vectors are capable of achieving long-term transgene expression in vitro and in vivo and are invaluable tools for manipulating gene expression in preclinical studies [11, 12].

Today, rAAV vectors are used for both overexpression and knockdown of specific genes in various tissues and cell-types. The cell-type specificity or “tropism” of AAV differs between serotypes and many serotypes display a high degree of tropism for nervous tissue. This CNS tropism coupled with the ability of rAAV to infect non-dividing, quiescent cells makes rAAV vectors ideal for pre-clinical neuroscience research. However, the widespread use of rAAV vectors is limited by the time and expense needed to produce them. The current methods for purifying rAAV utilize gradients of either iodixanol or cesium chloride [13–17]. These methods require the use of specialized centrifuges and expensive reagents which can prevent laboratories lacking the proper equipment or funding from producing rAAV in-house. Purified rAAV vectors can also be purchased from core facilities but this often takes several weeks and can be relatively expensive at ~$500–$2500 for a small-scale rAAV preparation. We have developed a method which overcomes these limitations by utilizing rAAV vectors secreted into the media following scalable PEI transfection of HEK293T cells. Most protocols purify rAAV vectors from the intracellular fraction but several groups have reported that rAAV is secreted into the media during production in HEK293 cells [18–20]. We demonstrate that this secreted rAAV can be utilized in lieu of purified virus for both in vitro and in vivo experiments without undergoing costly purification. As our laboratory and many others are currently utilizing rAAV vectors for CNS applications, we chose to assess the ability of secreted rAAVs to transduce CNS cells in vitro and in vivo. Only a few capsid pseudo-types were previously shown to be secreted so we examined the secretion of thirty different wild-type and engineered rAAVs (see Table 4). We show that unpurified preparations of secreted rAAVs from select pseudo-types can express transgenes in PNGC, BSC, and in vivo.

## Methods

### Construction of pseudo-typing plasmids for rAAV2/12, rAAV2/13, rAAV2/bovine, and rAAV2/avian

In order to clone new AAV pseudo-types, we used PCR to join the AAV2 Rep2 gene 3′ end with the capsid gene of AAV12, AAV13, Bovine AAV, or Avian AAV. The AAV2 3′ end of Rep2 and whole capsid 2 gene were cut out from the AAV2/9 helper plasmid using HindIII and EcoRV and replaced with the 3′-Rep2-AAV capsid gene for AAV12, AAV13, Avian-AAV, or Bovine-AAV.

### Preparation of PEI solution

This procedure follows the procedure set forth by [22]. Briefly, linear PEI (Polyscienes) was used as a 7.5 mM monomer aqueous stock solution and was diluted with de-ionized water at 0.323 g/l to make a final volume between 500 ml – 1 L (9 mg of the 50% (wt/vol) commercial solution). The solution was equilibrated to 70 C in a water bath for 1 h until all PEI particles were in solution. The solution was then adjusted to pH 8.0 with HCl and brought to the final volume. The solution was allocated into 50 ml conical tubes with 40 ml of solution in each tube. The tubes were frozen to − 80 C and then thawed to 37 C, and this cycle was repeated 3 times. The reagents were then stored at − 20 C until use.

### Human embryonic kidney cells (HEK293T)

The HEK293T cells were purchased from ATCC (ATCC CRL-3216). We used the ATCC recommended culturing method to maintain the cells: Dulbecco’s Modified Eagle’s Medium (DMEM), 10% Fetal Bovine Serum (FBS), 2 mM L-glutamine, and 1% Penicillin/Streptomycin.

### Production of secreted rAAV

HEK293T cells were plated in 2 ml media per well in 6-well plates 24 h before transfection. Cells were transfected with Polyethylenimine Linear (Polysciences) at 60–80% confluency using DNA ratios in Table 2. Production of rAAV2/1, 2/2, 2/5, 2/6, 2/7, and 2/8 required a double transfection of capsid-helper plasmid and transgene plasmid at a ratio of 3:1. Production of all other rAAVs required triple transfection of capsid-helper, adeno-helper, and transgene at a ratio of 1:2:1. Standard PEI transfection protocol was utilized. Sterile water, NaCl, and plasmid DNAs were combined in a total volume of 100 μl. In a separate tube, PEI solution was made containing PEI, NaCl, and water (Table 1). PEI solution was added (100 μl) to the DNA solution and incubated for 20 min at room temperature. The combined transfection reaction (200 μl) was pipetted drop-wise into the corresponding well. It is not necessary to change the media post-transfection. However, it is possible to change the media to a high-nutrient serum free media such as opti-MEM. Alternatively, if the DMEM or FBS can affect the viability of the cells to be transduced with rAAV, it can be replaced with the optimal media for the desired cell type. Media containing rAAV was harvested 72 h after transfection by centrifuging the media at 500 *g* for 5 min and collecting the supernatant. Media was aliquoted and frozen at -80 °C for further analysis.

**Table 1:** PEI transfection to produce rAAV in 6-well plate

AAV double transfection		AAV triple transfection		PEI solution	
Transgene plasmid	675 ng	Transgene plasmid	675 ng	PEI	30 μl
Capsid-helper plasmid	2025 ng	Capsid-helper plasmid	675 ng	1.5 M NaCl	10 μl
1.5 M NaCl	10 μl	Adeno helper plasmid	1350 ng	Water	60 μl
Water	to 100 μl	1.5 M NaCl	10 μl	Total	100 μl
Total	100 μl	Water	to 100 μl		
		Total	100 μl		

### Quantitative PCR analysis to determine genomic titer

Genomic titers were determined as previously described [23]. Briefly, viral preparations were treated with DNaseI (Life Technologies) to remove any residual DNA contaminants not present within intact virus. DNaseI was then inactivated through addition of 0.5 mM EDTA and incubation at 70 °C for 10 min. Viral DNA was then uncoated through digestion with Proteinase K (Life Technologies), followed by a second heat-inactivation at 95 °C for 10 min. Samples were compared against a standard curve of rAAV plasmid diluted from 1 × 10^3^ to 1 × 10^7^ copies per ml. The vector genomes per milliliter were calculated via quantitative real time PCR (qPCR). Custom probes were used (ThermoFisher cat# 4332078), which consisted of a pair of ITR PCR primers and a TaqMan probe with a dye label (FAM) on the 5′ end, a minor groove binder (MGB) and nonfluorescent quencher (NFQ) on the 3′ end. The ITR PCR forward primer was designed as 5′-GGAACCCCTAGTGATGGAGTT-3′ and the reverse primer as 5′-CGGCCTCAGTGAGCGA-3′. The qPCR reaction used TaqMan™ Fast Universal PCR Master Mix (ThermoFisher cat# 4352042) to make up the reaction along with the viral samples and probe. The samples were then run at: Initial denaturation cycle 95 °C for 30 s then a 39 cycle amplification of 95 °C for 5 s and 60 °C for 5 s.

### Production of purified intracellular rAAV preparations

HEK293T cells were split into two double-layer cell-stacks (Costar Product #3269, ThermoFisher Scientific, Waltham, MA) and maintained on DMEM containing 5% FBS in order to achieve 70–80% confluence at the time of transfection. Each cell-stack was co-transfected with endotoxin-free vector DNA (Qiagen, Valencia, CA) using Polyethylenimine Linear (PEI, Polysciences). For production of rAAV2/1-EGFP and rAAV2/8-EGFP each double-layer cell-stack was co-transfected with 90 μg of transgene plasmid and 270 μg of helper plasmid (pDP1rs or pDP8.ape). For production of rAAV2/9-EGFP and rAAV2/8-3Y-EGFP, each double-layer cell-stack was co-transfected with 90 μg of transgene plasmid, 90 μg of capsid helper plasmid, and 180 μg of adeno helper plasmid. At 72 h after transfection, cells were pelleted and lysed in the presence of 0.5% Sodium Deoxycholate and 50 U/ml Benzonase (Sigma) by repeated rounds of freeze/thaws at 80 °C and 50 °C. Viral particles were then isolated using a discontinuous Iodixanol gradient as previously described [24]. Samples were buffer exchanged to PBS using an Amicon Ultra filter 100,000 MWCO Centrifugation device (Millipore) into a final volume of ~ 200-μl sterile endotoxin–free phosphate-buffered saline (PBS). Genomic titers were obtained and rAAVs were aliquoted and stored at 80 °C. When needed, viruses were diluted in sterile 1X PBS, pH 7.2 and used immediately.

### Primary mixed Neuroglial cultures (PNGCs)

Primary mixed neuroglia cultures were prepared from the brains of postnatal day 0 (P0) B6/C3H mice (Envigo). Cerebral cortices were dissected from P0 mouse brains and dissociated in 2 mg/ml papain (Worthington) and 50 μg/mL DNAse I (Sigma) at 37 °C for 20 min. They were then washed three times in sterile Hank’s balanced salt solution (HBSS) to inactivate the papain and switched to 5% fetal bovine serum (HyClone) in Neurobasal-A growth media (Gibco), which includes 0.5 mM L-glutamine (ThermoFisher), 0.5 mM GlutaMax (ThermoFisher), 1% Penicillin/Streptomycin (ThermoFisher), and 0.02% SM1 supplement (Stemcell). The tissue mixture was then triturated three times using a 5 mL pipette followed by a Pasteur pipette, and strained through a 70 μm cell strainer. The cell mixture was then centrifuged at 200 *g* for 3 min, and re-suspended in fresh Neurobasal-A media. They were then plated onto poly-D-lysine coated 12 mm coverslips (Corning Life Sciences) submerged in 0.5 mL of media in a 24 well plate. Cells were maintained in the Neurobasal-A growth media mentioned above without fetal bovine serum (FBS) at 37 °C in a humidified 5% CO2 chamber.​ Unpurified media containing rAAV was applied directly into the culture medium on the fourth day of culture (4 DIV) at 1.0 × 10^10^ vector genomes per mL of culture media (final concentration of 0.5 × 10^10^ total vector genomes per well). This concentration was selected because we routinely use purified rAAV at 1.0 × 10^10^ vector genomes/mL of culture media to transduce PNGC. PNGCs were maintained with half media changes every 3 days until 10DIV, at which point they were fixed for imaging analysis.

### Imaging of primary Neuroglial cultures and brain slice cultures

PNGCs were fixed 4% paraformaldehyde for 10 min and coverslips were mounted on glass slides using Fluoromount-G with DAPI (Southern Biotech). BSCs were fixed with 4% paraformaldehyde for 1 h and mounted on glass slides using Fluoromount-G with DAPI (Southern Biotech). Images of EGFP fluorescence in PNGC and BSC were captured using a Keyence BZ-X700 all-in-one fluorescence microscope (Keyence Corp. of America) using the optical sectioning mode. Z-stacks were captured over 20 μm at recommended step-sizes and projected onto a full focus image using the BZ-analyzer.

### Quantification of transduction via EGFP expression

ImageJ was used to assess the total area of green fluorescence (pixel^2^) per visual field (mean ± SEM) as described previously [25, 26]. The area of green fluorescence was then normalized to the total area of DAPI fluorescence to control for variations in cell density. Images of each visual field were taken at 10X using the Keyence BZ-X700 all-in-one fluorescence microscope (Keyence Corp. of America).

### Biochemical extraction of PNGC lysates

PNGCs were plated into poly-D-lysine coated 6-well plates (Corning Life Sciences) in 2 mL of media per well. Cells were maintained in the Neurobasal-A growth media mentioned above without fetal bovine serum (FBS) at 37 °C in a humidified 5% CO2 chamber.​ Unpurified media containing rAAV or lysate purified rAAV was applied directly into the culture medium on the fourth day of culture (4 DIV) at 1.0 × 10^10^ vector genomes per mL of culture media (final concentration of 2.0 × 10^10^ total vector genomes per well). PNGCs were maintained with half media changes every 3 days until 10DIV, at which point they were harvested for western blot analysis. Each well was treated as an *n* = 1 and individually re-suspended in ice-cold PBS. PNGCs were then centrifuged at 500 x g for 3 minutes and PBS was removed from the cell pellet. Cell pellets were lysed in radio immunoprecipitation assay buffer containing 1% triton X-100 (Boston Bioproducts) and a cocktail of protease inhibitors (Sigma-Aldrich). Lysates were centrifuged at 1000 x g and soluble supernatants were subjected to western blot analysis.

### Immunofluorescent staining of PNGC

PNGCs were fixed in 4% paraformaldehyde for 10 min, washed with PBS, and blocked for 1 h at room temperature (RT) in PBS containing 5% FBS and 0.1% Triton X-100. Primary antibodies against GFAP (DAKO; 1:2000), Cd11b (ThermoFisher; 1:1000), MAP2 (Abcam; 1:1000), and MBP (BioRad; 1:1000) were diluted in PBS containing 5% FBS and incubated at RT for 2 h. PNGCs were then washed and Alexa Fluor labeled secondary antibodies (Invitrogen; 1∶1000) were added for 1 h. After washing with PBS, PNGCs were mounted on glass slides using Fluoromount-G with DAPI (Southern Biotech). Co-localization of EGFP expression and cell-type specific markers were captured using a Keyence BZ-X700 all-in-one fluorescence microscope (Keyence Corp. of America).

### Organotypic BSC

Organotypic BSCs were prepared from postnatal day 8–9 B6/C3H mice (Envigo) as previously described [27]. Unpurified media containing rAAV was applied directly into the culture medium on the first day of culture (0 DIV) at 1.0 × 10^10^ vector genomes per mL of culture media (final concentration of 1.0 × 10^10^ total vector genomes per well containing three BSCs). This number of vector genomes has been utilized in previous studies to transduce BSCs with purified rAAV [28]. Slices for EGFP expression were maintained with media changes every 3–4 days until 14DIV, at which point they were fixed for imaging analysis. Slices for tau expression were maintained with media changes every 3–4 days until 28DIV, at which point they were harvested for biochemical fractionation or fixed for thioflavin S staining.

### Biochemical extraction of BSC lysates

Slice cultures for assessment of insoluble tau from three wells (nine slices in total) were harvested into ice-cold PBS and presented as *n* = 1. Slices were washed in PBS, pelleted, and sarkosyl extractions were performed as previously described [29]. Slice cultures for comparison of lysate purified and unpurified media preparations from three wells (nine slices in total) were harvested into ice-cold PBS and presented as *n* = 1. Slices were lysed in radio immunoprecipitation assay buffer containing 1% triton X-100 (Boston Bioproducts) and a cocktail of protease inhibitors (Sigma-Aldrich). Lysates were centrifuged at 1000 x g and soluble supernatants were subjected to western blot analysis.

### SDS-PAGE and immunoblotting

Protein concentrations were determined by BCA assay (Thermo Scientific) using bovine serum albumin (BSA) as the standard. SDS sample buffer was added and 5 μg protein from the sarkosyl insoluble fraction and 15 μg of protein from the low-speed supernatant was separated on 4–12% SDS-PAGE gels (Bio-Rad) and electrophoretically transferred to polyvinylidene difluoride membranes, as described previously [30]. Membranes were blocked in 0.5% casein for 1 h and incubated overnight at 4 °C submerged in 0.5% casein containing primary antibodies against total human tau (CP27; 1:500), tau phosphorylated at S396/404 (PHF-1; 1:500), or GAPDH (abcam; 1:5000). Membranes were then washed three times with TBS and incubated for 1 h with fluorophore conjugated secondary antibodies Alexa Fluor 680 anti-mouse IgG (Thermo Fisher Scientific; 1:10000) and IRDye 800 goat anti-rabbit IgG (Li-Cor Biosciences; 1:10000). Membranes were then washed three times with TBS and protein bands were detected using the multiplex Li-Cor Odyssey Infrared Imaging system (Li-Cor Biosciences).

### Western blot quantification and statistical analysis

Relative band intensity was quantified using the multiplex Li-Cor Odyssey Infrared system imaging software (Li-Cor Biosciences). Values for EGFP (Invitrogen; 1∶1000) were normalized to bands corresponding to β-actin obtained from the same individual sample (Sigma-Aldrich; 1:5000). The EGFP/actin ratio was calculated for each experimental group of cultures transduced with either unpurified media preparations or lysate-purified preparations of the same rAAV pseudo-type. The values obtained from the group transduced with unpurified media were then expressed as percent of control of the average values of the lysate-purified group. Data is presented as the percent control ± SEM and statistical comparisons between the two groups for each pseudo-type were conducted using a student’s un-paired T-test.

### Thioflavin S staining

Organotypic BSCs were washed in PBS, fixed on their inserts with 4% paraformaldehyde for 1 h, and then stained with 0.0125% Thioflavin S as previously described [31]. In brief, a stock solution of 1% Thioflavin S (Sigma-Aldrich) in 50% EtOH in PBS was filtered through a 0.2-μm filter. Individual slices (*n* = 1) were cut from their membranes and autofluorescence reagent (Millipore) was applied for 5 min, and then slices were washed in 40% ethanol (EtOH). BSCs were incubated with 0.0125% Thioflavin S in 50% EtOH in PBS for 3 min in the dark, followed by 50% EtOH and PBS washes. BSCs were then mounted on slides with Fluoromount-G (Southern Biotech) and imaged to identify any amyloidogenic β-sheet structures in these sections using the Keyence BZ-X700 all-in-one fluorescence microscope (Keyence Corp. of America).

### Mice and neonatal injections

All animal procedures were approved by the Institutional Animal Care and Use Committee at the University of Florida. All animals were housed three to five to a cage and maintained on ad libitum food and water with a 12 h light/dark cycle. Intracerebroventricular injections of rAAVs were carried out in nontransgenic neonatal mice (B6/C3H, Envigo, Indianapolis, IN) on day P0 as described previously [23]. Two microliters of unpurified media containing rAAV were administered bilaterally for a total of four microliters per animal. Media preparations were injected without dilution in order to assess the maximum possible transduction for each capsid. Genomic titers of each rAAV media preparation used for injection and total number of vector genomes injected can be found below in Table 2. After thirty days, mice were euthanized by trans-cardiac perfusion, brains harvested, and fixed overnight in 4% paraformaldehyde solution at 4 °C. Brains were transferred to PBS before processing and paraffin embedding.

**Table 2:** Titers of rAAVs used for in vivo injection

rAAV Name	genomic titer vg/mL	total genomes injected per ventricle
rAAV2/1	1.6E+ 10	3.1E+ 07
rAAV2/2	1.5E+ 09	3.0E+ 06
rAAV2/3	9.5E+ 10	1.9E+ 08
rAAV2/4	5.9E+ 10	1.2E+ 08
rAAV2/5	7.0E+ 08	1.4E+ 06
rAAV2/6	8.4E+ 10	1.7E+ 08
rAAV2/7	3.5E+ 10	6.9E+ 07
rAAV2/8	5.2E+ 10	1.0E+ 08
rAAV2/9	6.3E+ 10	1.3E+ 08
rAAV2/Rh10	3.6E+ 10	7.2E+ 07
rAAV2/12	8.4E+ 09	1.7E+ 07
rAAV2/13	2.6E+ 10	5.2E+ 07
rAAV2/Bovine	4.2E+ 10	8.4E+ 07
rAAV2/Avian	3.2E+ 10	6.4E+ 07
rAAV2/1-2Y + T	1.1E+ 11	2.2E+ 08
rAAV2/2-3Y	1.2E+ 11	2.4E+ 08
rAAV2/2-4Y	1.4E+ 11	2.8E+ 08
rAAV2/2-5Y	6.8E+ 10	1.4E+ 08
rAAV2/2-4Y + T + K	1.7E+ 11	3.3E+ 08
rAAV2/5-Y	3.9E+ 10	7.8E+ 07
rAAV2/5-2Y	1.4E+ 11	2.8E+ 08
rAAV2/6-2S	2.4E+ 11	4.7E+ 08
rAAV2/6-T + S	1.1E+ 11	2.2E+ 08
rAAV2/6-2Y + T	1.4E+ 11	2.9E+ 08
rAAV2/8-Y	2.0E+ 11	3.9E+ 08
rAAV2/8-2Y	2.4E+ 11	4.8E+ 08
rAAV2/8-3Y	5.6E+ 10	1.1E+ 08
rAAV2/8-2Y + T	1.7E+ 11	3.3E+ 08
rAAV2/9-PHP.B	7.4E+ 10	1.5E+ 08
rAAV2/9-PHP.eB	1.8E+ 11	3.6E+ 08

### Immunohistochemical staining of paraffin embedded tissue

Brains were divided into coronal sections, paraffin embedded, cut to a thickness of 5 μm, and attached to glass slides (Fisher Scientific). Immunohistochemistry was performed using primary antibodies against EGFP (Invitrogen; 1∶500), GFAP (DAKO; 1:2000), or IBA-1 (WAKO; 1:2000), followed by development using ImmPress polymer detection reagents (Vector Labs). Immunohistochemically stained sections were captured using the Scanscope XT image scanner (Aperio). Double immunofluorescent staining was performed using primary antibodies against EGFP (JL-8, Clontech; 1∶1000), GFAP (DAKO; 1:2000), IBA-1 (WAKO; 1:2000), MAP2 (Abcam; 1:1000) and developed using Alexa Fluor labeled secondary antibodies (Invitrogen; 1∶500). Fluorescently labeled sections were captured using a Keyence BZ-X700 all-in-one fluorescence microscope (Keyence Corp. of America).

### Additional concentration to obtain high titer rAAV

HEK293T cells were plated in 20 mL of DMEM containing 10%FBS and 1% Pen/Strep in a 15 cm plate 24 h before transfection. Cells were transfected with Polyethylenimine Linear (Polysciences) at 80% confluency with the DNA ratios in Table 3. Sterile water, NaCl, and plasmid DNAs were combined in a total volume of 1 mL. In a separate tube, PEI solution was made containing PEI, NaCl, and water. PEI solution was added (1 mL) to the DNA solution and incubated for 20 min at room temperature. The combined transfection reaction (2 mL) was pipetted drop-wise into the corresponding plate. 24 h after transfection, all media was removed and replaced with 20 mL of opti-MEM containing 1% Pen/Strep without FBS. Cells were then returned to the incubator for an additional 48 h before harvest. 72 h after transfection, media was removed and placed in a sterile 50 mL conical centrifuge tube. Media was then subjected to centrifugation at 500 *g* for 5 min to remove cell debris and supernatant was collected. 200 μl of the media was saved to determine the titer of rAAV before concentration. The remaining media was pipetted onto a 15 mL Amicon Centrifugal Filter Unit with a 100,000 Da molecular weight cutoff (Millipore Sigma). The filter unit was then subjected to centrifugation at 2000 *g* in 10 min intervals at room temperature. After each 10 min spin, the unit was visually inspected to determine the level of media remaining in the insert. This method was repeated until the remaining media was below the 250 μL mark on the insert. This (100X) concentrated media containing rAAV was then transferred to a sterile 1.5 mL tube and briefly vortexed to ensure homogeneity. The concentrated rAAV media was then titered, aliquoted, and stored at -80 °C.

**Table 3:** PEI Transfection to produce concentrated rAAV preparations

AAV double transfection		AAV triple transfection		PEI solution	
Transgene plasmid	12.5 μg	Transgene plasmid	12.5 μg	PEI	0.5 mL
Capsid-helper plasmid	37.5 μg	Capsid-helper plasmid	25 μg	1.5 M NaCl	0.1 mL
1.5 M NaCl	0.1 mL	Adeno helper plasmid	12.5 μg	Water	0.4 mL
Water	to 1 mL	1.5 M NaCl	0.1 mL	Total	1 mL
Total	1 mL	Water	to 1 mL		
		Total	1 mL		

## Results

### Transient transfection of HEK293T cells produces rAAV2/1 in the media

**Fig. 1:**
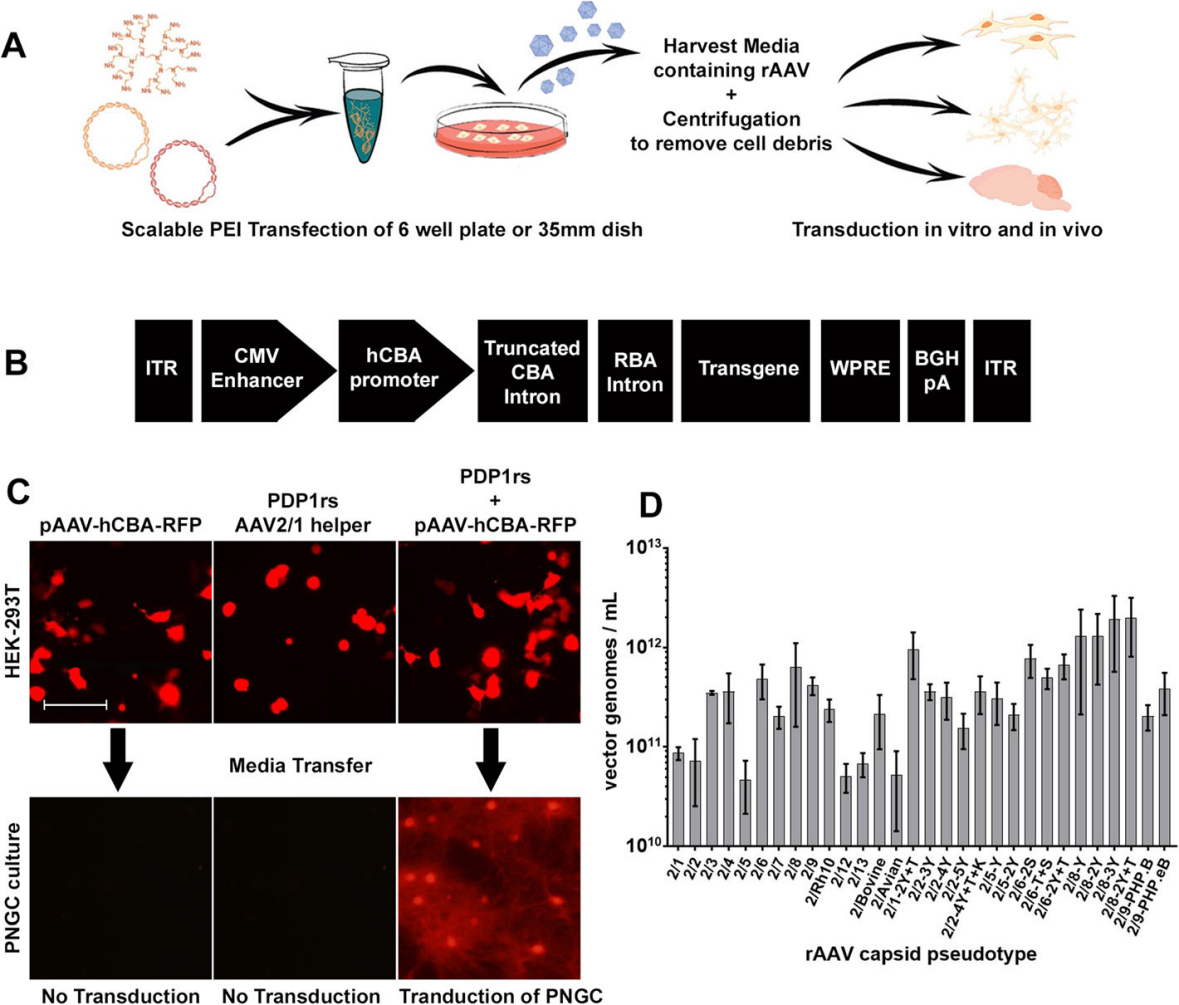
Scalable PEI transfection of HEK293T cells produces secreted rAAV. **a** Schematic diagram of method for production of secreted rAAV. **b** Single-stranded (ss) rAAV2 vectors used in this study contained the cytomegalovirus (CMV) enhancer, hybrid chicken β-actin (hCBA) promoter, truncated CBA intron, rabbit beta-globin splice acceptor, woodchuck hepatitis virus post-transcriptional regulatory element (WPRE), bovine growth hormone polyadenylation signal, and transgene encoding either red fluorescent protein (TurboRFP) or enhanced green fluorescent protein (EGFP). **c** Only co-transfection of both rAAV2/1 capsid helper (pDP.rs) and transgene (pAAV-hCBA-RFP) produces rAAV in the media capable of transducing PNGC. Bar, 100 μm **d** Genomic titers of all secreted rAAVs at 72 h post-transfection quantified as vector genomes/mL of culture media (Mean ± SEM *N* = 3)

We first sought to determine if rAAV secreted during HEK293T cell-based production could be used to transduce primary cultures without purification. We transfected HEK293T cells with the transgene plasmid pAAV-hCBA-TurboRFP (Fig. 1b), the AAV1 capsid helper plasmid pDP1.rs, or both plasmids together. Only co-transfection of both transgene and AAV helper plasmids (Fig. 1a) should produce rAAV in the media. In order to test this, we transferred culture media from transfected HEK293T cells to PNGC and assessed RFP expression seven days later. Only media from cells co-transfected with both plasmids produced RFP expression in PNGC (Fig. 1c). Media from cells transfected with only a single plasmid did not elicit RFP expression in PNGC, indicating that PEI and plasmid DNA alone in the absence of rAAV does not induce transgene expression. These data demonstrate that the media contains infectious rAAV vectors.

### All rAAV pseudo-types are secreted during virus production

After successfully producing media containing infectious rAAV2/1 vectors, we next sought to determine which other rAAV pseudo-types are secreted during virus production. HEK293T cells were co-transfected with pAAV-hCBA-EGFP plasmid (Fig. 1b) and each of the thirty rAAV capsid helper plasmids (Table 4). Genomic titers were assessed in three separate media preparations of each capsid. We compared the average titer for each pseudo-type (Fig. 1d) and discovered ~ 40-fold variance from a low ~ 4.7 × 10^10^ vector genomes (vg)/mL for rAAV2/5-EGFP to a high of ~ 2.0 × 10^12^ vg/mL for a capsid mutant of rAAV2/8-EGFP (Y447F-Y733F). These data demonstrate that the genomic titers of rAAV present in the media vary widely and are consistent with previous data suggesting that secretion of the various capsids is quite distinct.

**Table 4:** List of rAAVs with average titers in the media

rAAV Name	Mutations	Average titer in Media (vg/mL)	Source
rAAV2/1		8.7E+ 10	Plasmid Factory
rAAV2/2		7.2E+ 10	Plasmid Factory
rAAV2/3		3.5E+ 11	National Gene Vector Biorepository
rAAV2/4		3.6E+ 11	National Gene Vector Biorepository
rAAV2/5		4.7E+ 10	PlasmidFactory
rAAV2/6		4.9E+ 11	Plasmid Factory
rAAV2/7		2.0E+ 11	Provided by Sergei Zolotukhin, Ph.D., University of Florida
rAAV2/8		6.3E+ 11	PlasmidFactory
rAAV2/9		4.2E+ 11	Provided by Sergei Zolotukhin, Ph.D., University of Florida
rAAV2/Rh10		2.4E+ 11	Provided by Christian Mueller, Ph.D., University of Massachusetts
rAAV2/12		5.1E+ 10	Capsid gene provided by John A. Chiorini, Ph.D., NIH
rAAV2/13		6.8E+ 10	Capsid gene provided by John A. Chiorini, Ph.D., NIH
rAAV2/Bovine		2.1E+ 11	Capsid gene provided by John A. Chiorini, Ph.D., NIH
rAAV2/Avian		5.2E+ 10	Capsid gene provided by John A. Chiorini, Ph.D., NIH
rAAV2/1-2Y + T	T492V + Y705F + Y731F	9.5E+ 11	Provided by Arun Srivastava, Ph.D., University of Florida
rAAV2/2-3Y	Y444F + Y500F + Y730F	3.6E+ 11	Provided by Shannon E. Boye, PH.D., University of Florida
rAAV2/2-4Y	Y272F + Y444F + Y500F + Y730F	3.2E+ 11	Provided by Shannon E. Boye, PH.D., University of Florida
rAAV2/2-5Y	Y272F + Y444F + Y500F + Y704F + Y730F	1.5E+ 11	Provided by Shannon E. Boye, PH.D., University of Florida
rAAV2/2-4Y + T + K	Y272F + Y444F + Y500F + Y730F + T491V + K556R	3.6E+ 11	Provided by Shannon E. Boye, PH.D., University of Florida
rAAV2/5-Y	Y719F	2.1E+ 11	Provided by Shannon E. Boye, PH.D., University of Florida
rAAV2/5-2Y	Y263F + Y719F	3.1E+ 11	Provided by Shannon E. Boye, PH.D., University of Florida
rAAV2/6-T + S	T492V + S663V	7.8E+ 11	Provided by Arun Srivastava, Ph.D., University of Florida
rAAV2/6-2Y + T	T492V + Y705F + Y731F	5.0E+ 11	Provided by Arun Srivastava, Ph.D., University of Florida
rAAV2/6-2S	S663V + S551V	6.7E+ 11	Provided by Arun Srivastava, Ph.D., University of Florida
rAAV2/8-Y	Y733F	2.0E+ 12	Provided by Shannon E. Boye, PH.D., University of Florida
rAAV2/8-2Y	Y447F + Y733F	2.0E+ 12	Provided by Shannon E. Boye, PH.D., University of Florida
rAAV2/8-3Y	Y275F + Y447F + Y733F	1.3E+ 12	Provided by Shannon E. Boye, PH.D., University of Florida
rAAV2/8-2Y + T	Y447F + T494V + Y733F	1.3E+ 12	Provided by Shannon E. Boye, PH.D., University of Florida
rAAV2/9-PHP.B	Insertion at AA588 (TLAVPFKA)	2.0E+ 11	Synthesized from Genscript based on reference (21)
rAAV2/9-PHP.eB	A587D + Q588G and insertion at AA588 (TLAVPFKA)	3.8E+ 11	Synthesized from Genscript based on reference (21)

**Table 5:** rAAV titers before and after media concentration

Construct / Preparation #	Non-concentrated titer vg/mL	100X concentrated titer vg/mL
rAAV2/8-EGFP-Prep #1	2.8E+ 11	3.4E+ 13
rAAV2/8-EGFP-Prep #2	7.0E+ 10	2.5E+ 12
rAAV2/8-EGFP-Prep #3	5.5E+ 11	4.8E+ 13
rAAV2/8-3Y-EGFP-Prep #1	2.5E+ 12	1.3E+ 14
rAAV2/8-3Y-EGFP-Prep #2	1.8E+ 12	2.1E+ 14
rAAV2/8-3Y-EGFP-Prep #3	6.1E+ 11	1.7E+ 13

### Efficient transduction of PNGC by media containing secreted rAAV

**Fig. 2:**
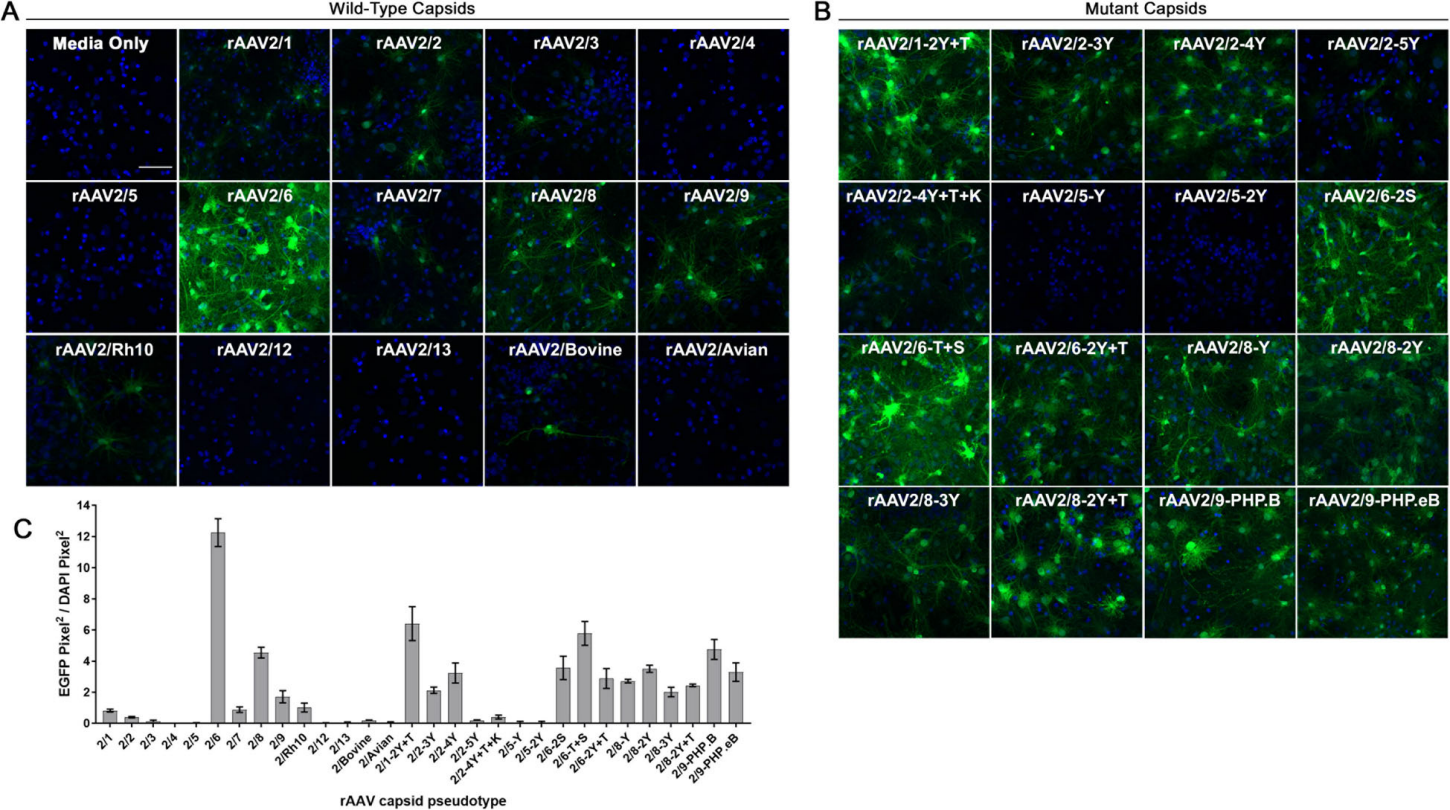
Efficient transduction of PNGC by rAAV secreted into the media. Primary mixed neuroglial cultures (PNGCs) were transduced at 3 DIV with rAAV2-EGFP vectors packaged in thirty different capsid serotypes (1 × 10^10^ vg/mL of PNC culture media). At 10 DIV, cultures were fixed and EGFP expression was imaged. Representative images of PNC transduction by rAAV2-EGFP vectors pseudo-typed with wild-type capsids (**a**) or mutant capsids (**b**) Bar, 50 μm. Images were taken with the same exposure time to directly compare transduction efficiency (**c**) Quantification of transduction efficiency in PNC (Mean ± SEM *n* = 3)

These rAAV vector preparations were then tested for their ability to transduce PNGCs. All preparations were added at 1.0 × 10^10^ vector genomes per mL of PNGC media and EGFP expression was evaluated 7 days later. Representative images and quantification of EGFP fluorescence are shown in Fig. 2. For the wild-type capsids (Fig. 2a), PNGCs were efficiently transduced by rAAV 2/6, 2/8, and 2/9 but were inefficiently transduced by rAAV2/1, 2/2, 2/3, 2/4, 2/5, 2/7, 2/Rh10, 2/12, 2/13, 2/Bovine, and 2/Avian. All mutant capsids (Fig. 2b) efficiently transduced PNGC except rAAV2/2-5Y, 2/2-4Y + T + K, 2/5-Y, and 2/5-2Y. Quantification of EGFP fluorescence revealed that wild-type rAAV2/6 achieved the highest levels of EGFP expression relative to all other wild-type and mutant capsid pseudo-types (Fig. 2c). As images were captured at the same intensity to directly compare transduction efficiency, some pseudo-types appeared to have no EGFP expression. In order to determine if any transduction could be detected for these capsids, images were captured at higher exposure (Fig. S1). This analysis revealed that low levels of EGFP could be detected at higher exposure in cells transduced by rAAV2/4, 2/5, 2/12, 2/13, 2/5-Y, and 2/5-2Y, while rAAV2/avian showed no transduction. Next, we sought to determine which CNS cell-types were transduced in the PNGC cultures by co-localizing EGFP expression with markers for neurons, astrocytes, oligodendrocytes, and microglia. We chose to investigate the cell-types transduced by three of the most efficient rAAV preparations (rAAV2/6, 2/8, and 2/1-2Y + T) and one less efficient preparation (rAAV2/1). All four rAAVs were able to transduce neurons, astrocytes, oligodendrocytes, and microglia (Fig. S2). This was as predicted because the hCBA promoter is not cell-type specific and should drive EGFP expression in all transduced cells [32]. Notably, we did not observe differences in the cell types transduced by rAAV2/1 and mutant rAAV2/1-2Y + T, suggesting that the increased transduction efficiency was likely not due to alterations in cellular tropism. These differences are possibly due to the capsid mutations which were previously shown to prevent degradation and increase the amount of rAAV that reaches the nucleus [33–37]. We also assessed whether unpurified rAAV preparations can induce transgene expression levels comparable to those of iodixanol purified rAAV preparations. Lysate purified or unpurified media preparations of the rAAV2-hCBA-EGFP vector packaged in capsids 1, 8, and 9 were added to PNGCs at 1.0 × 10^10^ vector genomes per mL of PNGC media. Western blotting for EGFP in PNGC lysates was used to directly compare expression levels between purified and unpurified preparations of the same capsid pseudo-type (Fig. S3). Densitometry with statistical analysis did not find a significant difference between EGFP levels in PNGCs transduced with purified or unpurified rAAV2/1 and 2/9. We did find significantly higher (~ 25%) EGFP levels in PNGC cultures transduced with unpurified rAAV2/8 when compared to the purified preparation. These results suggest that unpurified preparations could potentially be utilized in lieu of purified virus to induce similar levels of transgene expression in PNGC.

### Transduction of BSC by select capsids

**Fig. 3:**
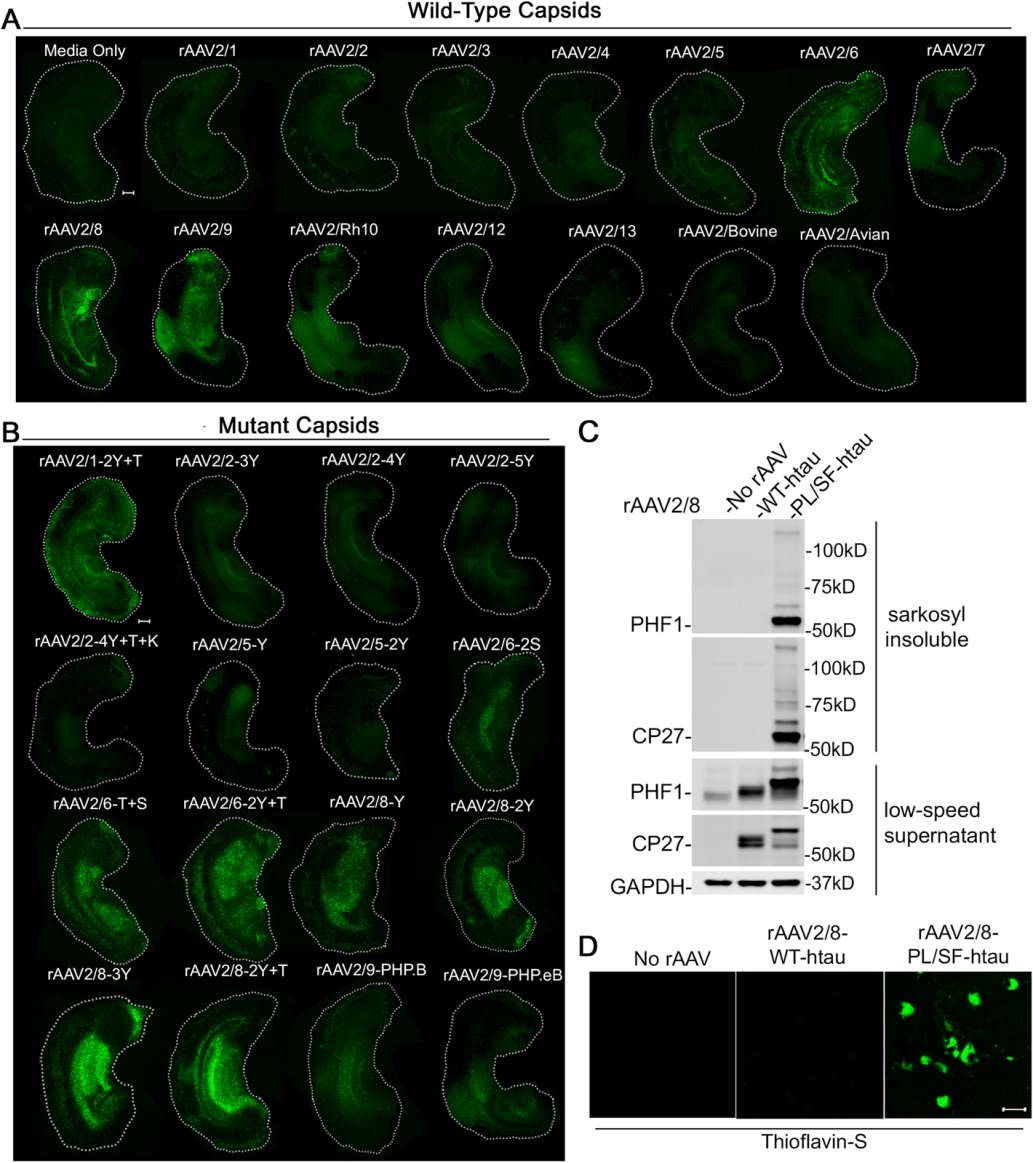
Transduction of BSC by secreted rAAV from select capsids. Organotypic BSCs were prepared and transduced at 0 DIV with rAAV (1 × 10^10^ vg/mL of BSC culture media). BSCs were maintained in culture until 14 DIV, fixed, and fluorescence of EGFP throughout the BSC was imaged. Representative images of BSC transduction by rAAV2-EGFP vectors pseudotyped with wild-type capsids (**a**) or mutant capsids (**b**) Bar, 100 μm. Images were taken with the same exposure time to directly compare transduction efficiency. **c** BSCs were transduced with rAAV2/8-WT-htau or rAAV2/8-P301L-S320F-htau under the hCBA promoter (1 × 10^10^ vg/mL of BSC culture media) at 0 DIV and maintained in culture until 28 DIV. Lysates were sequentially extracted and immunoblotted for total human tau (CP27), tau phosphorylated at Ser396/404 (PHF-1), and GAPDH as a loading control. Representative western blots of tau in the low-speed supernatant and sarkosyl insoluble fraction are shown. **e** Transduced slice cultures were fixed, stained with 0.0125% Thioflavin S, and imaged to identify any β-sheet structures in these sections Bar, 25 μm

In order to determine whether unpurified rAAV vector preparations can transduce cultures that are more recapitulative of the in vivo brain, we assessed transduction in three-dimensional mouse brain slice cultures (BSCs) derived from the cortex and hippocampus of non-transgenic mice. We have previously shown that lysate purified rAAV2/1, 2/2, 2/6, 2/8, and 2/9 transduce BSCs efficiently [28]. We wanted to extend these findings by assessing whether unpurified preparations of secreted rAAV can similarly transduce BSCs. With the exception of rAAV2/8, secreted rAAVs of other wild-type and mutant capsids have not been assessed for their ability to transduce BSCs. We utilized our method to survey thirty different rAAV preparations to determine which capsids were capable of transducing BSCs. BSCs were prepared from postnatal day 8 (P8) mice and transduced at 0 DIV with each of the thirty unpurified preparations. Viruses were diluted so that 1.0 × 10^10^ vector genomes was added to each well containing three slices. As the multiple cellular layers of brain slice cultures can interfere with quantification of fluorescence, we qualitatively assessed EGFP fluorescence at 14 DIV in three individual slices for each of the thirty capsids. Images showing EGFP fluorescence in a single, representative slice are shown in Fig. 3a (wild-type capsids) and Fig. 3b (mutant capsids). We observed robust EGFP expression in BSCs transduced with media preparations of rAAV2/6, 2/8, 2/9, 2/Rh10, 2/1-2Y + T, 2/6-2S, 2/6-T + S, 2/6-2Y + T, 2/8-Y, 2/8-2Y, 2/8-3Y, 2/8-2Y + T, 2/9-PHP.B, and 2/9-PHP.eB. Limited EGFP fluorescence was observed in BSCs transduced with rAAV2/1, 2/2, 2/3, 2/4, 2/5, 2/7, 2/12, 2/13, 2/Bovine, 2/Avian, and all mutants of rAAV2/2 and 2/5. We also assessed whether the EGFP expression levels induced by unpurified preparations in BSC were comparable to that of purified preparations. As we routinely utilize lysate purified rAAV2/8 for our BSC experiments, we compared unpurified and purified preparations of rAAV2/8 via western blotting for EGFP (Fig. S4). Quantification confirmed that EGFP levels were not significantly different in BSCs transduced with unpurified or purified preparations of rAAV2/8-hCBA-EGFP added at the same titer. We then sought to determine if unpurified rAAV preparations could drive expression of non-EGFP transgenes in BSC. Previous studies in our lab have utilized lysate-purified rAAV2/8 to express the human microtubule associated protein tau (MAPT) in BSC in order to model pathological changes found in Alzheimer’s Disease [28]. Expression of tau with both disease associated mutations P301L and S320F was shown to result in the formation of thioflavin S positive neurofibrillary tangles (NFTs) comprised of highly phosphorylated, sarkosyl-insoluble tau. In the current study we attempted to recapitulate these findings using unpurified media preparations of rAAV2/8. We packaged both wild-type and P301L/S320F human tau in rAAV2/8 under the hCBA promoter and collected the media containing rAAV. Addition of rAAV-containing media at 1.0 × 10^10^ vg/mL resulted in significant overexpression of both wild-type and mutant tau detected via western blot (Fig. 3c). In line with our previous findings, wild-type tau was phosphorylated but remained soluble, while expression of P301L/S320F tau produced hyper-phosphorylated, sarkosyl-insoluble tau. In addition, only P301L/S320F tau formed thioflavin S positive NFTs after 28 days (Fig. 3d). These data demonstrate the potential utility of unpurified rAAV for expressing physiologically relevant transgenes in BSC.

### In vivo transduction via somatic brain transgenesis

**Fig. 4:**
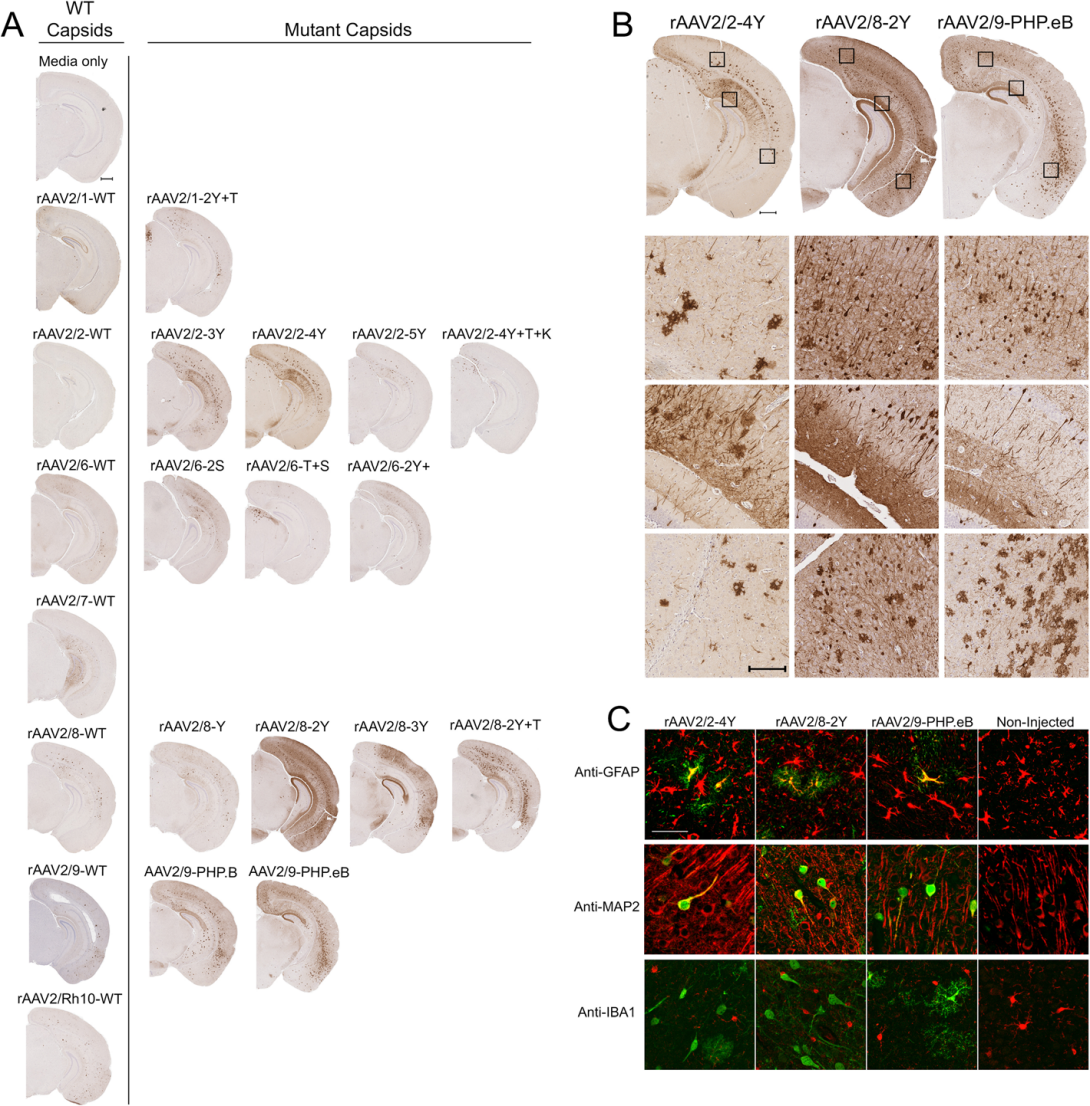
In vivo transduction via somatic brain transgenesis. Media containing rAAV2-EGFP vectors was injected directly into each lateral ventricle of non-transgenic mice at postnatal day 0 (P0). **a** Representative brain sections from mice aged P30 stained with an anti-EGFP antibody (*n* = 3–5). Bar, 500 μm. **b** Higher magnification (10X) images show transduction by select mutant rAAV2/2, 2/8, and 2/9 capsids. Bar, 150 μm (**c**) Immuno-fluorescent co-staining for EGFP expression (green) and cell-type specific markers (red) reveals transduction of astrocytes (anti-GFAP) and neurons (anti-MAP2) but not microglia (anti-IBA1) Bar, 50 μm. Additional co-localization data can be found in Fig. S3

After demonstrating that unpurified media viruses efficiently transduce in vitro, we next assessed their ability to transduce CNS cells in vivo. Intracerebroventricular (ICV) injection of rAAV at postnatal day 0 (P0) has been shown to result in widespread transgene expression in the mouse CNS [23]. In the current study, we injected undiluted media containing rAAV directly into each lateral ventricle of non-transgenic mice at postnatal day 0 (P0) and assessed EGFP expression in the brain after thirty days. Three to four animals were injected with each preparation and representative images of EGFP expression in a single animal are shown in Fig. 4a. We detected abundant EGFP positive cells in the cortex and hippocampus of mice injected with rAAV2/2-3Y, 2/2-4Y, 2/8-Y, 2/8-2Y, 2/8-3Y, 2/8-2Y + T, 2/9 PHP.B, and 2/9-PHP.eB. Figure 4b shows higher magnification images of EGFP staining in the cortex and hippocampus from mice injected with unpurified preparations of rAAV2/2-4Y, rAAV2/8-2Y, and rAAV2/9-PHP.eB. In vivo transduction among the thirty rAAV capsids was not a direct comparison, as media containing unpurified rAAV was injected without dilution to assess the maximum possible transduction for each preparation. Variation in the number of injected vector genomes also may have contributed to the observed differences in transduction. This data can serve as a starting point for other laboratories seeking to optimize this method for their individual application. Genomic titers of each injected rAAV media preparation can be found in Table 2. We also investigated the in vivo cell-type specificity of rAAV2/2-4Y, rAAV2/8-2Y, and rAAV2/9-PHP.eB by co-localizing with neuronal, astrocytic, and microglial markers (Fig. 4c and Fig. S5). We observed all three preparations to transduce both neurons and astrocytes in vivo but not microglia. We also compared the in vivo transduction of unpurified media preparations to lysate-purified rAAV2/8-3Y injected at the same genomic titer. Three animals were bilaterally injected with 2.0 × 10^8^ vector genomes and representative images of EGFP expression in a single animal are shown (Fig. S6). We observed similar transduction in mice injected with either unpurified or purified preparations, suggesting that unpurified preparations could potentially be utilized in lieu of purified virus for select in vivo applications.

### Expression of mIL6 and mIL10 in vivo

**Fig. 5:**
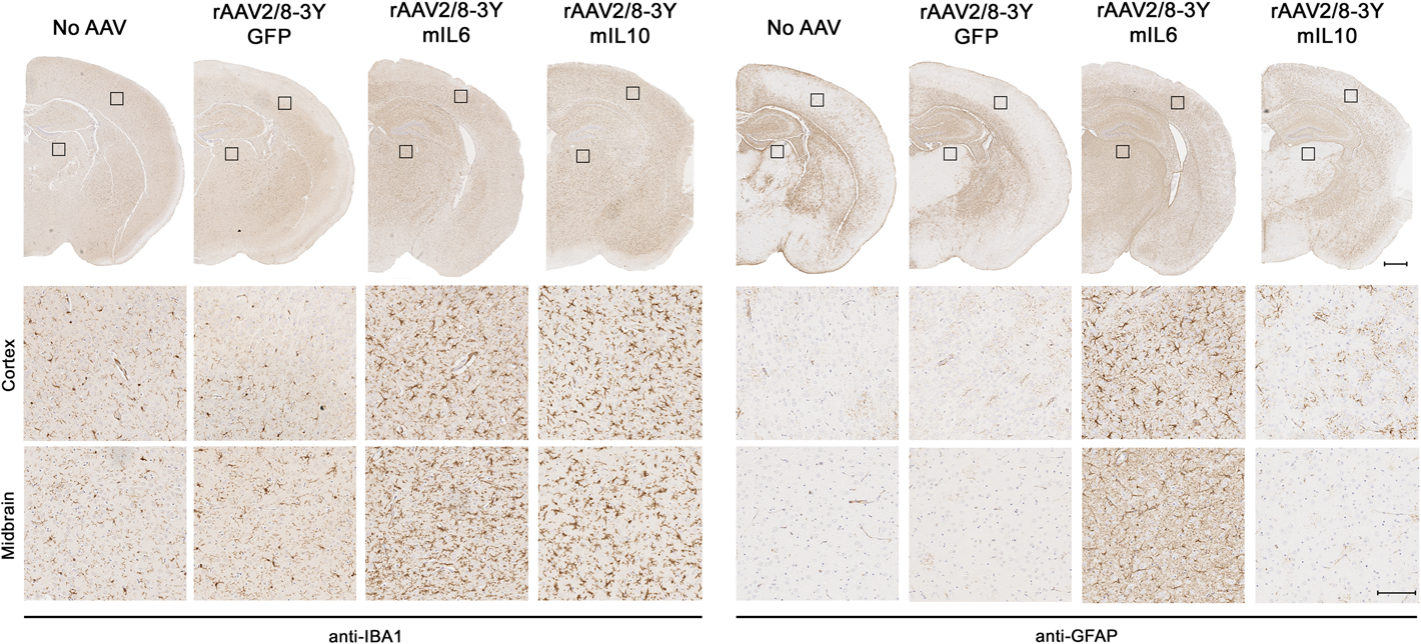
rAAV-mediated expression of mIL6 and mIL10 in vivo. Media containing either rAAV2/8-3Y-EGFP, mIL6, or mIL10 was injected directly into each lateral ventricle of non-transgenic mice at P0. (**a**) Representative brain sections from mice aged P30 stained with an anti-IBA1 antibody to assess microgliosis (**a**) or an anti-GFAP antibody to assess astrogliosis (**b**). Brains injected with media containing no rAAV or rAAV2/8-3Y-EGFP were used as controls. Bar, 500 μm (hemi-brain) or 100 μm (10X magnification)

After demonstrating media preparations of mutant rAAV can induce widespread EGFP expression in the brain, we next evaluated whether these viruses can express non-EGFP transgenes at sufficient levels to induce a biological response. To do this, we packaged rAAV vectors encoding the mouse cytokines interleukin-6 (mIL6) and interleukin-10 (mIL10) into the rAAV2/8-3Y mutant capsid and injected the rAAV-containing media into p0 non-transgenic mice using the same ICV paradigm. Our results showed that injection of media containing rAAV-mIL6 induced extensive microgliosis (Fig. 5a) and astrogliosis (Fig. 5b). Injection of rAAV-mIL10 also increased microgliosis and altered astrocytic morphology, while injection of rAAV-EGFP or media alone did not alter the morphology of either cell type. The observed phenotypes were entirely consistent with previous studies in our lab using rAAV-mIL6 and mIL10 to alter immunoproteostasis in transgenic models of amyloid-beta deposition [38, 39]. This suggests that secreted rAAV in the media can be utilized without purification to induce features of neurodegenerative disease similar to those achieved with lysate-purified rAAV.

### Additional concentration to obtain titers from 10^13^ to 10^14^ vg/mL

Our data indicates that small-scale transfections can produce rAAV in the media at concentrations of 10^10^–10^12^ (vg)/ml. Although this is sufficient for many applications, some experimental paradigms may only allow for a few microliters of rAAV to be utilized such as transduction in a 96-well plate or ICV injection. In order to overcome this limitation, we developed a method for one-step concentration of media virus to obtain unpurified rAAV preparations in the range of 10^13^–10^14^ vg/mL vector genomes (vg) per mL that are typical of purified rAAV preparations. HEK293T cells in a single 15 cm dish were transfected, changed over to serum free media, and allowed to secrete rAAV for approximately 48 h. This media was then concentrated 100X using a centrifugal filter. The resulting titers for concentrated rAAV2/8 and rAAV2/8-3Y-EGFP were indeed increased ~ 100 fold to be in the range of 10^12^–10^14^ vg/mL (Table 5). We then tested whether 1 μL of 100X concentrated rAAV2/8-EGFP media at 1.0 × 10^13^ vg/mL could produce comparable PNGC transduction as 100 μL of non-concentrated rAAV2/8-EGFP media at 1.0 × 10^11^ vg/mL. Our results show that concentrated media containing rAAV added at ~ 100 fold less volume produces similar transduction when compared to non-concentrated media (Fig. S7). This data indicates that concentration of media is a viable strategy for rapid and cost-effective production of minimally purified high titer rAAV preparations.

## Discussion

The current rAAV vectors are versatile tools for manipulating gene expression both in vitro and in vivo. Nevertheless, a limitation to the widespread use of rAAV is the time and resources needed to produce purified rAAV preparations. We sought to address this limitation by investigating whether secreted rAAV vectors present in the media following small-scale transfection of HEK293T cells could be utilized to express transgenes without undergoing costly purification. As a laboratory who routinely utilizes rAAV vectors, we had access to thirty different rAAV capsids including both wild-type and engineered variants. Packaging rAAV2-EGFP vectors in each of the thirty capsids revealed large differences in secretion levels and transduction of CNS cells. We found that media preparations containing secreted rAAVs packaged in select capsids are capable of transducing mouse primary mixed neuroglial cultures (PNGCs), ex-vivo brain-slice cultures (BSCs), and the mouse CNS in vivo.

Our results indicate that select capsid serotypes produce robust transduction depending on the experimental paradigm, while others achieve only modest transduction across all paradigms. It is important to note that transduction was assessed at a single vector concentration and that the efficiency of some capsids might be increased by utilizing higher vector concentrations. It may be necessary to optimize unpurified rAAV preparations for a given application by testing different combinations of vector concentration, capsid pseudo-type, and promoter. This study expressed transgenes under the ubiquitous hCBA promoter but cell-type specific expression could be achieved using widely available rAAV vectors with alternative promoters. In addition, unpurified rAAVs could be utilized to rapidly assess a panel of transgenes or evaluate the tropism of novel capsids. Although we only assessed transduction of CNS cells, our method could also be used to screen rAAV capsids for the ability to transduce non-CNS cell-types.

These results also suggest that the tropism of secreted rAAVs may not always mirror that of intracellular rAAVs of the same pseudo-type. Some secreted rAAVs were less effective at transducing CNS cells than would be expected based on known tropisms such as rAAV2/5. In our study, wild-type and mutant rAAV2/5 media preparations produced minimal transduction in CNS-derived cultures when added at the same genomic titer as other rAAVs. These findings are in contrast to studies utilizing lysate-purified rAAV2/5 to efficiently transduce the mammalian CNS [11]. One possible explanation is that secreted viral particles may be inherently different than those found intracellularly. Several recent papers have identified secreted rAAV particles inside vesicular bodies such as exosomes [40–47]. The secreted rAAVs utilized in our study may be associated with exosomes and this association could potentially alter the way in which they interact with the target cell. Future studies will need to characterize which pseudo-types associate with exosomes and the degree to which this association contributes to transduction efficiency. For our purposes, we did not find it imperative to assess whether the secreted rAAV in our media preparations exist within exosomes. Our method produces media containing rAAV capable of in vitro and in vivo transduction, regardless of the factors which contribute to this efficiency. Although our study is the first to directly compare the secretion and transduction of thirty different capsid pseudo-types, other groups have indeed developed protocols for isolating rAAV from HEK293 culture media. One method isolates exosomal rAAV using ultracentrifugation of media obtained during rAAV production [43]. This strategy does have advantages over gradient-based purification of intracellular rAAV but still requires large volumes of media and ultracentrifuges that may be cost-prohibitive to smaller laboratories. Another method purifies rAAV from the media via polyethylene glycol precipitation with subsequent aqueous two-phase partitioning [48]. This method can be completed without an ultracentrifuge or iodixanol gradient but still requires several additional time-consuming steps such as media changes and incubations with chemical reagents. The advantage of our method is that rAAV can be produced in 72 h and is ready for use on the same day as the harvest without any additional manipulation. In addition, other methods have only been applied to media derived from a few select pseudo-types so it is unknown whether they are applicable to all rAAV vectors.

## Conclusions

We find that high-titer secreted rAAV can be obtained without specialized equipment for only the cost of a small-scale PEI transfection and that rAAV media preparations can be used across several CNS applications. In some cases, the rAAV obtained using this method may be completely sufficient, reducing the need for purified virus. In others, small-scale preparations could allow for the rapid optimization of vectors before scaling up for large-scale purification. We confirm that unpurified media preparations of rAAV from select pseudo-types are capable of inducing EGFP expression levels comparable to that of lysate purified preparation. We also use unpurified rAAV preparations to model both tau pathology in BSC and neuro-inflammation in vivo, indicating that expression levels of non-EGFP transgenes is sufficient to induce biologic effects. This method should enable the cost-effective screening of transgene variants such as disease-associated mutations in proteins such as tau without needing to produce purified virus for each variant. In addition, it could potentially be utilized to determine the optimal capsid/promoter combinations or evaluate the tropism of novel capsids. Overall, this method should allow for rapid hypothesis testing and provide any laboratory access to the versatile rAAV tool kit.

## Supplementary information

**Additional file 1: Figure S1**. Imaging PNC at higher exposure reveals inefficient transduction by select capsids. Several capsids did not show detectable EGFP expression when PNC transduction was assessed at the same exposure for all capsids. Imaging at a higher exposure revealed that all of these capsids were indeed able to inefficiently transduce PNC except rAAV2/avian Bar, 100 μm.

**Additional file 2: Figure S2.** Co-localization of EGFP expression in PNGC with cell type-specific markers. EGFP expression in PNGC (green) was co-localized with immuno-fluorescent staining of cell-type specific markers (red) for astrocytes (anti-GFAP), neurons (anti-MAP2), oligodendrocytes (anti-MBP), and microglia (anti-Cd11b) Bar, 50 μm.

**Additional file 3: Figure S3**. EGFP expression in PNGC cultures transduced with titer-matched unpurified media or lysate purified rAAV packaged in select capsids. PNGCs were transduced with either unpurified media rAAV or lysate purified rAAV of capsids rAAV2/1, 2/8, and 2/9. All preparations were diluted to 1 × 10^10^ vg/mL of PNGC culture media to allow for direct comparison of EGFP levels. (A) PNGC lysates were immunoblotted for both EGFP and actin. Western blots used for quantification are shown (*n* = 3). (B-D) Bar charts show EGFP signal normalized to actin and expressed as percent of control wells transduced with lysate purified rAAV packaged of the same pseudo-type. Data are mean ± SEM. *N* = 3 student’s un-paired t-test **p = 0.0073, ns = not significant.

**Additional file 4: Figure S4**. EGFP expression in BSC cultures transduced by unpurified media preparations compared to lysate purified preparations of rAAV2/8. BSCs were transduced with either unpurified media rAAV or lysate purified rAAV of capsids rAAV2/8. All preparations were diluted to 1 × 10^10^ vg/mL of BSC culture media to allow for direct comparison of EGFP levels. (A) BSC lysates were immunoblotted for both EGFP and actin. Western blots used for quantification are shown (*n* = 3). (B) Bar chart shows EGFP signal normalized to actin and expressed as percent of control wells transduced with lysate purified rAAV. Data are mean ± SEM. *N* = 3 student’s un-paired t-test ns = not significant.

**Additional file 5: Figure S5**. In vivo co-localization of EGFP expression with cell type-specific markers. Immuno-fluorescent co-staining of EGFP expression (green) and cell-type specific markers (red) for astrocytes (anti-GFAP), neurons (anti-MAP2), and microglia (anti-IBA1) Bar, 50 μm.

**Additional file 6: Figure S6**. In vivo EGFP expression in mice injected with unpurified media or lysate-purified preparations of rAAV2/8-3Y. Unpurified media or lysate-purified was injected directly into each lateral ventricle of non-transgenic mice at postnatal day 0 (P0). Representative brain sections from mice aged P30 stained with an anti-EGFP antibody are shown (*n* = 3). Bar, 500 μm (hemi-brain), 100 μm (higher-magnification).

**Additional file 7: Figure S7**. Additional concentration to obtain titers from 10^13^ to 10^14^ vg/mL. HEK293T cells in a 15 cm dish were transfected to produce either rAAV2/8-EGFP or rAAV2/8-3Y-EGFP and allowed to secrete rAAV into serum free media for 48 h. Media was concentrated 100X and transduction of PNGC was compared to that of non-concentrated media containing rAAV. Similar levels of transduction were observed 7 days after addition of either 100 μL of non-concentrated rAAV2/8-EGFP at ~ 1.0 × 10^11^ or 1 μL of 100X concentrated rAAV2/8-EGFP at ~ 1.0 × 10^13^. Bar, 50 μm.

## Data Availability

The datasets used and/or analyzed during the current study are available from the corresponding author on reasonable request.

## Abbreviations

BSC: Brain slice culture
CNS: Central nervous system
EGFP: Enhanced green fluorescent protein
mIL10: Mouse interleukin 10
mIL6: Mouse interleukin 6
NFT: Neurofibrillary tangles
PNGC: Primary mixed neuroglial cultures
rAAV: Recombinant adeno-associated virus
RFP: Red fluorescent protein
SBT: Somatic brain transgenesis
