# Supplementary figures and images for: Utilizing minimally purified secreted rAAV for rapid and cost-effective manipulation of gene expression in the CNS
Source: Mol Neurodegener. 2020 Mar 2;15:15. doi: 10.1186/s13024-020-00361-z (PMC7053119; doi:10.1186/s13024-020-00361-z)

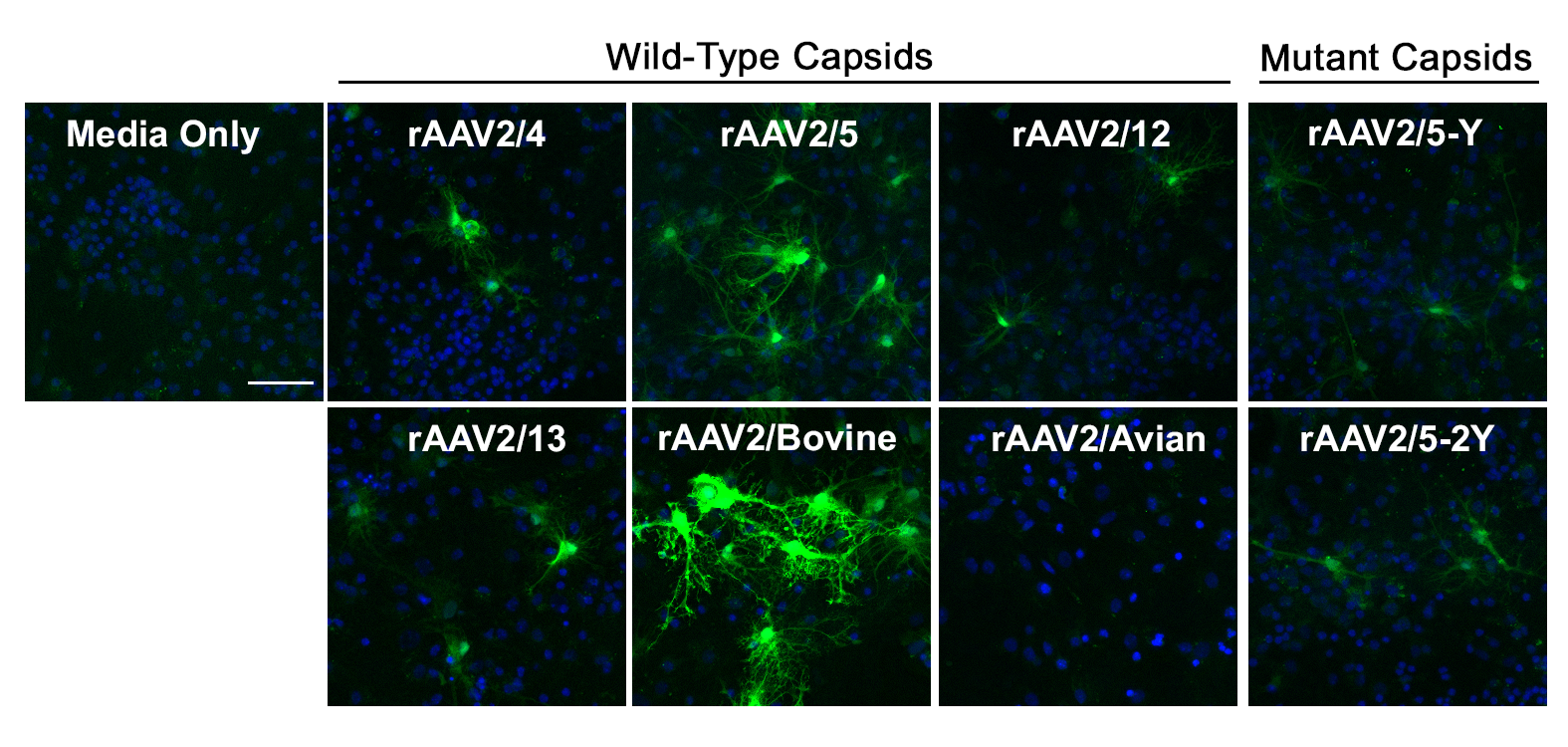

Supplement: Supplementary file 1 — Additional file 1: Figure S1. Imaging PNC at higher exposure reveals inefficient transduction by select capsids. Several capsids did not show detectable EGFP expression when PNC transduction was assessed at the same exposure for all capsids. Imaging at a higher exposure revealed that all of these capsids were indeed able to inefficiently transduce PNC except rAAV2/avian Bar, 100 μm. [file 13024_2020_361_MOESM1_ESM.tif]

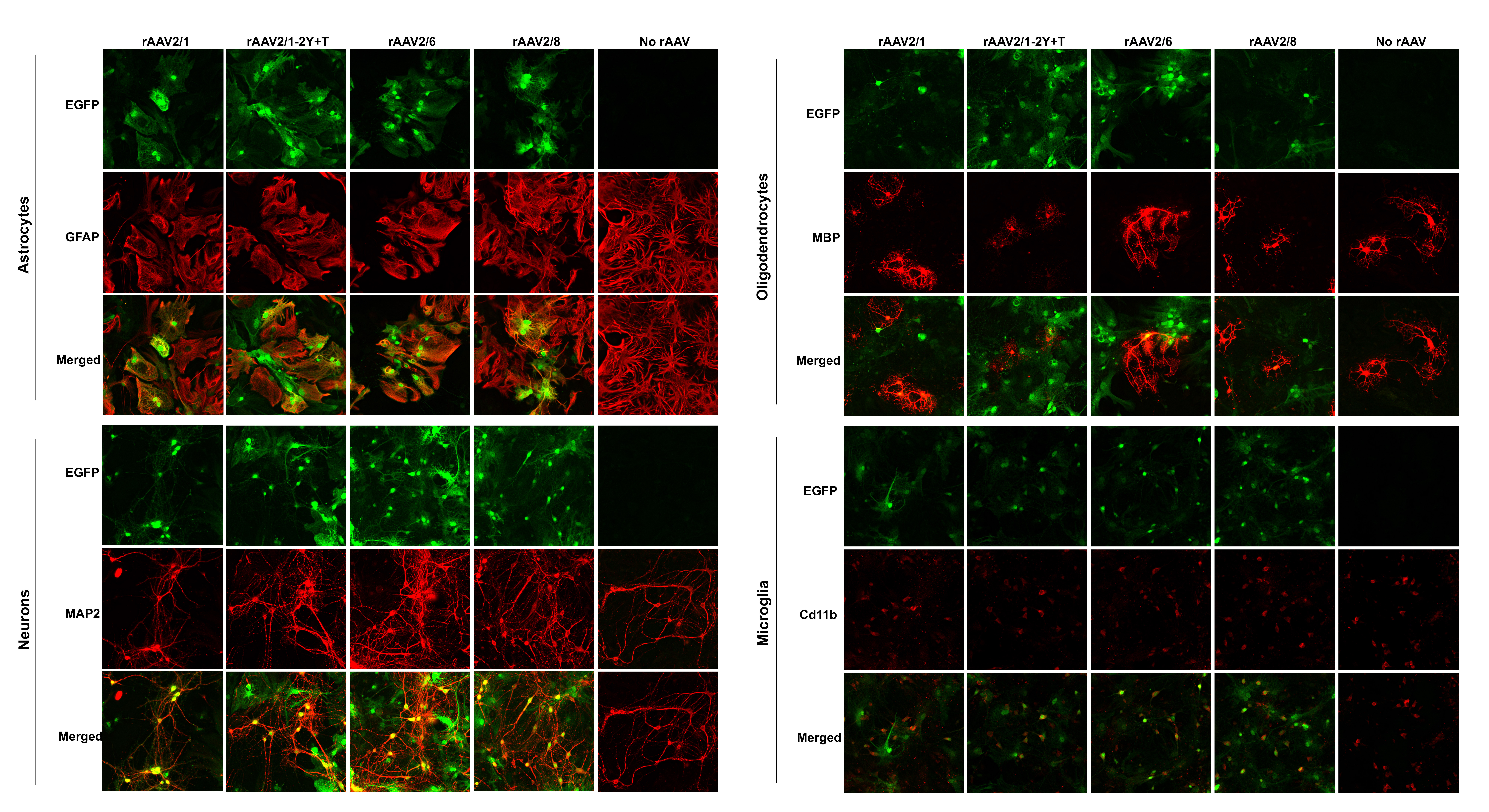

Supplement: Supplementary file 2 — Additional file 2: Figure S2. Co-localization of EGFP expression in PNGC with cell type-specific markers. EGFP expression in PNGC (green) was co-localized with immuno-fluorescent staining of cell-type specific markers (red) for astrocytes (anti-GFAP), neurons (anti-MAP2), oligodendrocytes (anti-MBP), and microglia (anti-Cd11b) Bar, 50 μm. [file 13024_2020_361_MOESM2_ESM.jpg]

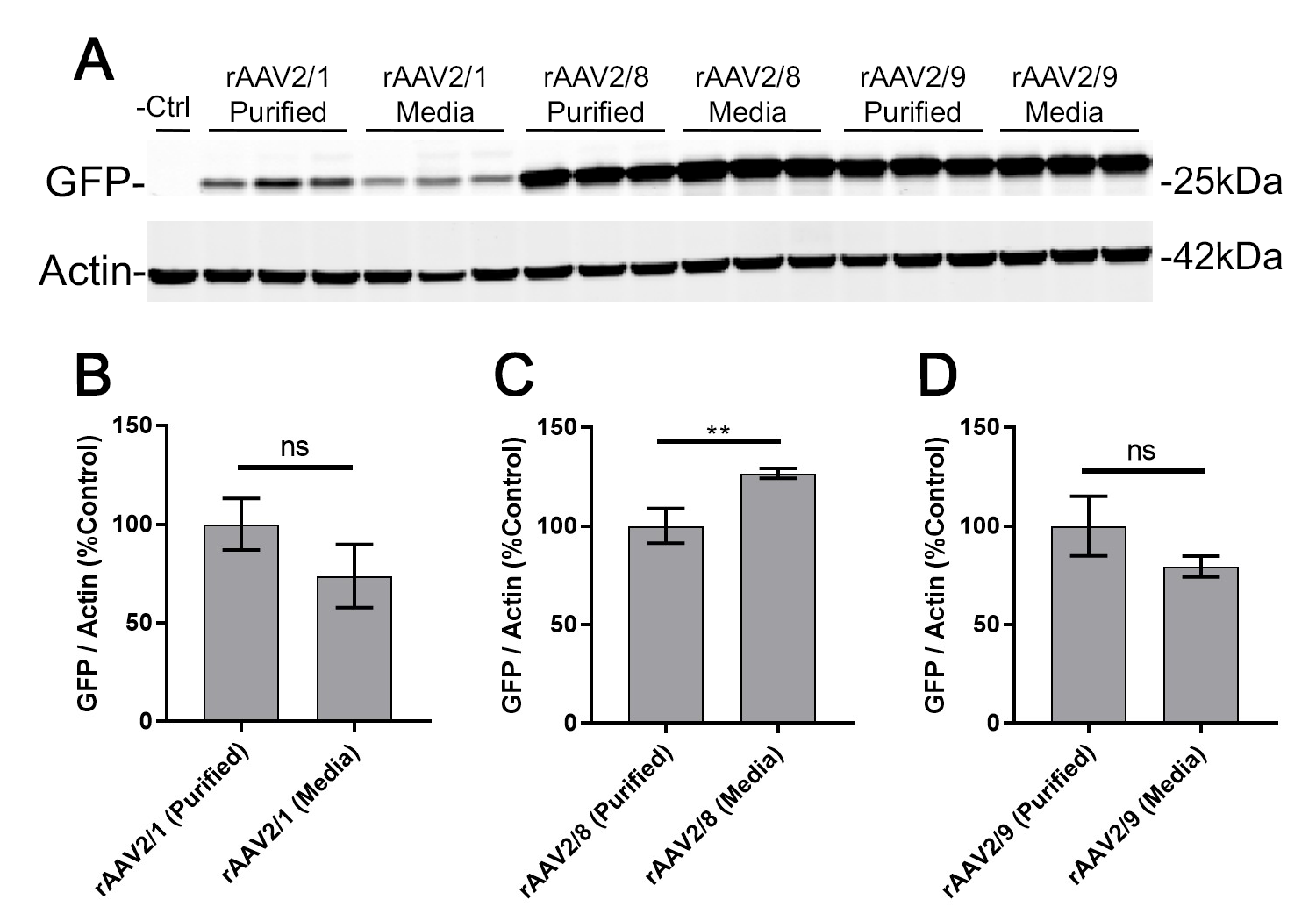

Supplement: Supplementary file 3 — Additional file 3: Figure S3. EGFP expression in PNGC cultures transduced with titer-matched unpurified media or lysate purified rAAV packaged in select capsids. PNGCs were transduced with either unpurified media rAAV or lysate purified rAAV of capsids rAAV2/1, 2/8, and 2/9. All preparations were diluted to 1 × 10^10^ vg/mL of PNGC culture media to allow for direct comparison of EGFP levels. (A) PNGC lysates were immunoblotted for both EGFP and actin. Western blots used for quantification are shown (n = 3). (B-D) Bar charts show EGFP signal normalized to actin and expressed as percent of control wells transduced with lysate purified rAAV packaged of the same pseudo-type. Data are mean ± SEM. N = 3 student’s un-paired t-test **p = 0.0073, ns = not significant. [file 13024_2020_361_MOESM3_ESM.tif]

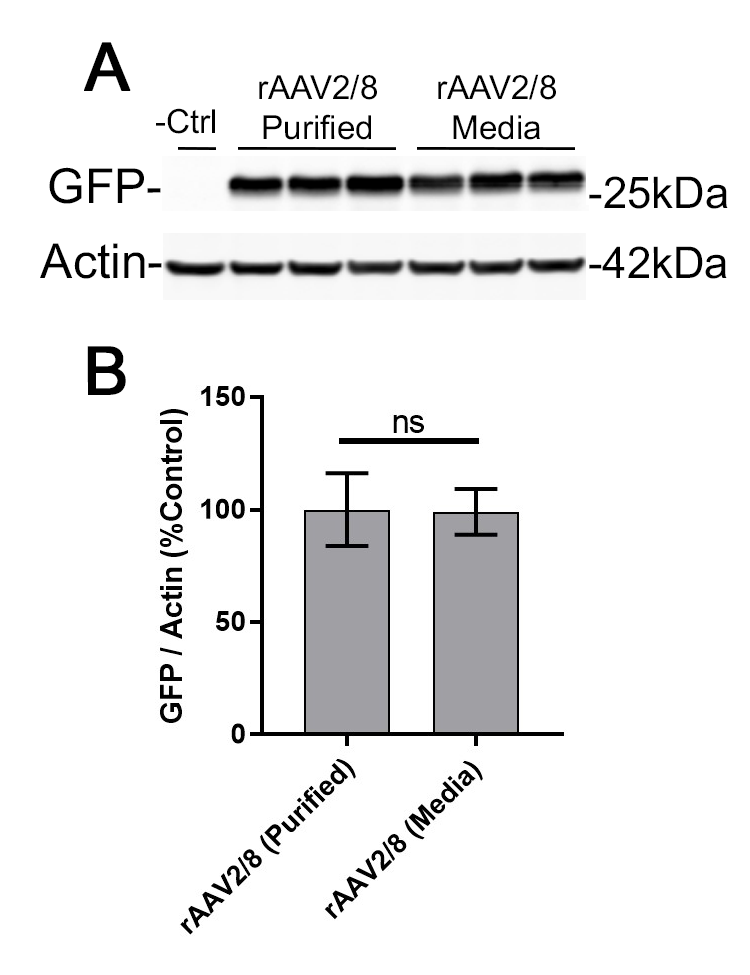

Supplement: Supplementary file 4 — Additional file 4: Figure S4. EGFP expression in BSC cultures transduced by unpurified media preparations compared to lysate purified preparations of rAAV2/8. BSCs were transduced with either unpurified media rAAV or lysate purified rAAV of capsids rAAV2/8. All preparations were diluted to 1 × 10^10^ vg/mL of BSC culture media to allow for direct comparison of EGFP levels. (A) BSC lysates were immunoblotted for both EGFP and actin. Western blots used for quantification are shown (n = 3). (B) Bar chart shows EGFP signal normalized to actin and expressed as percent of control wells transduced with lysate purified rAAV. Data are mean ± SEM. N = 3 student’s un-paired t-test ns = not significant. [file 13024_2020_361_MOESM4_ESM.tif]

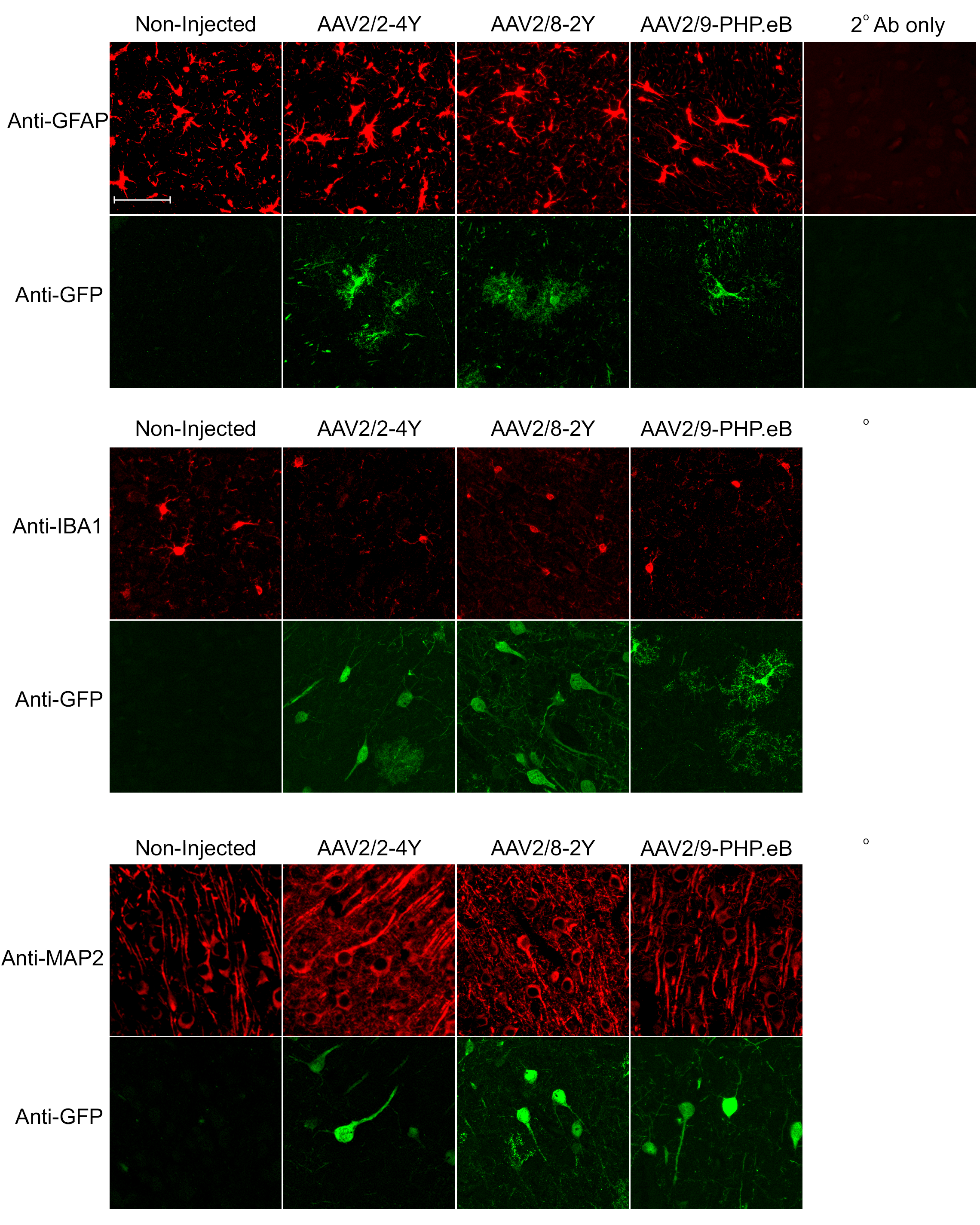

Supplement: Supplementary file 5 — Additional file 5: Figure S5. In vivo co-localization of EGFP expression with cell type-specific markers. Immuno-fluorescent co-staining of EGFP expression (green) and cell-type specific markers (red) for astrocytes (anti-GFAP), neurons (anti-MAP2), and microglia (anti-IBA1) Bar, 50 μm. [file 13024_2020_361_MOESM5_ESM.tif]

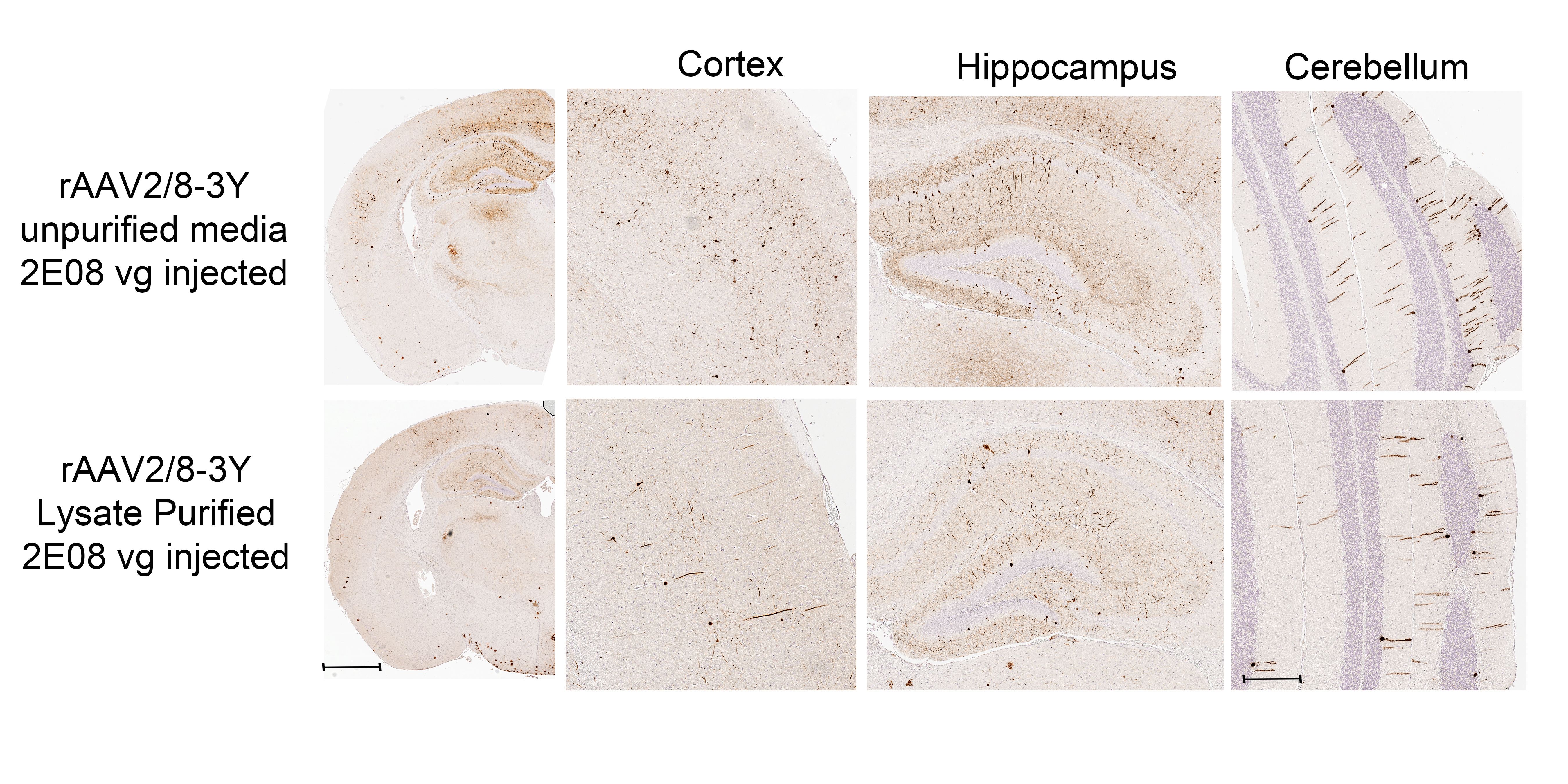

Supplement: Supplementary file 6 — Additional file 6: Figure S6. In vivo EGFP expression in mice injected with unpurified media or lysate-purified preparations of rAAV2/8-3Y. Unpurified media or lysate-purified was injected directly into each lateral ventricle of non-transgenic mice at postnatal day 0 (P0). Representative brain sections from mice aged P30 stained with an anti-EGFP antibody are shown (n = 3). Bar, 500 μm (hemi-brain), 100 μm (higher-magnification). [file 13024_2020_361_MOESM6_ESM.jpg]

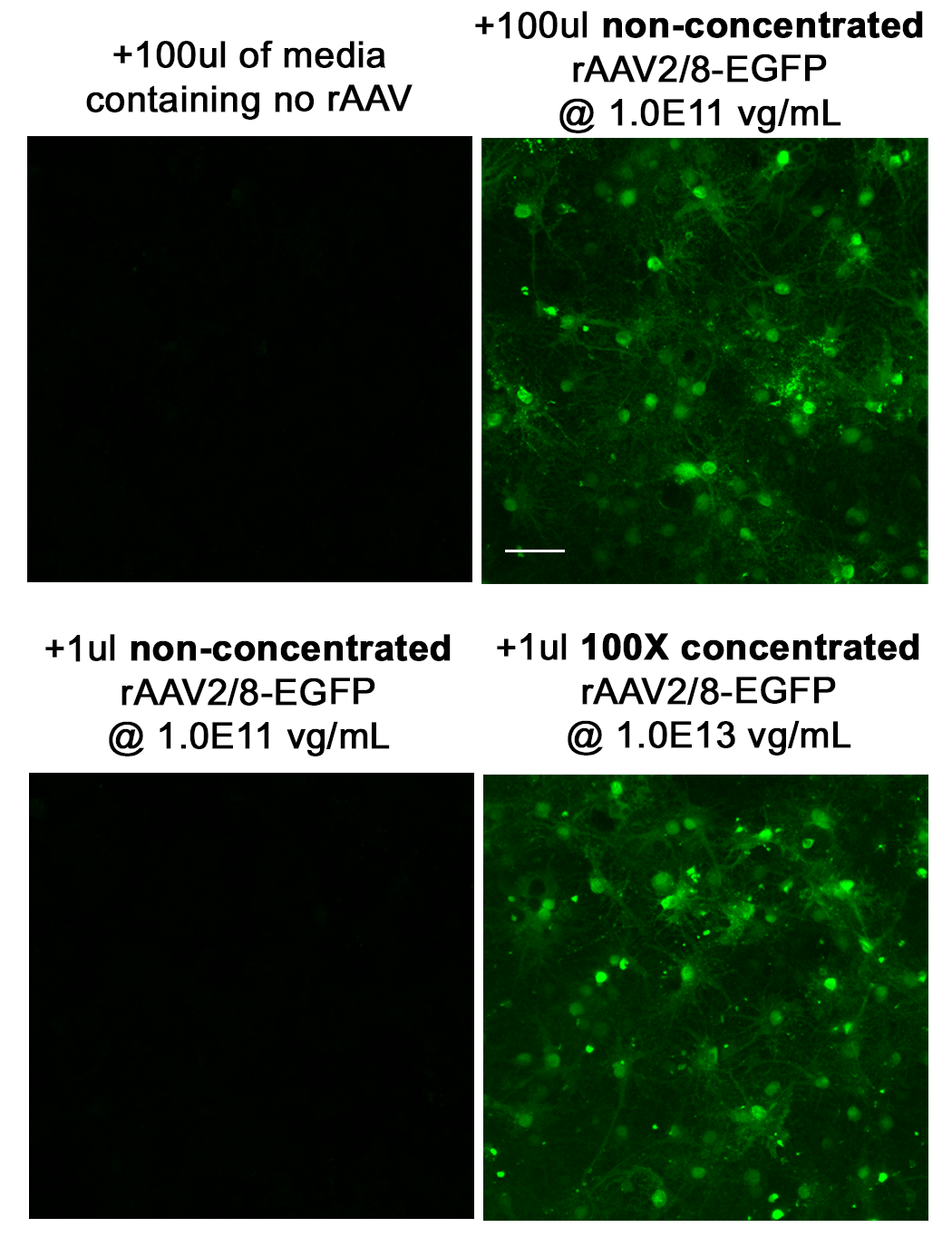

Supplement: Supplementary file 7 — Additional file 7: Figure S7. Additional concentration to obtain titers from 1013 to 1014 vg/mL. HEK293T cells in a 15 cm dish were transfected to produce either rAAV2/8-EGFP or rAAV2/8-3Y-EGFP and allowed to secrete rAAV into serum free media for 48 h. Media was concentrated 100X and transduction of PNGC was compared to that of non-concentrated media containing rAAV. Similar levels of transduction were observed 7 days after addition of either 100 μL of non-concentrated rAAV2/8-EGFP at ~ 1.0 × 10^11^ or 1 μL of 100X concentrated rAAV2/8-EGFP at ~ 1.0 × 10^13^. Bar, 50 μm. [file 13024_2020_361_MOESM7_ESM.tif]
